# An Ethnobotanical Study of Medicinal Plants in Kelala District, South Wollo Zone of Amhara Region, Northeastern Ethiopia

**DOI:** 10.1155/2021/6651922

**Published:** 2021-02-24

**Authors:** Yimer Assen, Mesfin Woldearegay, Abeba Haile

**Affiliations:** Department of Biology, College of Natural and Computational Sciences, Debre Birhan University, P.O. Box 445, Debre Birhan, Ethiopia

## Abstract

This research was carried out to record and document the medicinal plants and associated indigenous plant use knowledge of the local people in Kelala District of Amhara Region, Ethiopia. Ethnobotanical data were collected by conducting preprepared semistructured interview items with 60 informants. Focus group discussion and guided field walk were also used. Data were analyzed using basic analytical tools and descriptive statistics. Determination of informant consensus factor, fidelity level, and ranking was performed. A total of 82 medicinal plants distributed in 79 genera and 45 families were collected. Of these plants, 43 species were used to treat human ailments, and 33 species were used to treat livestock ailments and the remaining 6 species were used to treat both human and livestock ailments. The majority of medicinal plants were harvested from the wild environments. The family Solanaceae occupied the first rank with seven species followed by Fabaceae, Asteraceae, Cucurbitaceae, Apiaceae, and Euphorbiaceae with four species each. The most frequently used plant parts were leaves (42.2%) followed by seeds (15.2%), roots (8.1%), and fruits (7.6%). Freshly harvested plant parts (72.68%) were mostly used for remedy preparation compared to dried forms (24.74%) whereas crushing, which accounted for 41.12%, and powdering (24.37%) were the most widely used methods of remedy preparation in the study area. Expansion of farmlands by cutting trees heavily threatens medicinal plants and therefore needs due attention. High ranking medicinal plants are good candidates for further research in drug discovery and development.

## 1. Introduction

Plants have a long history of being used for medicinal purposes. About 80% of the developing world's population still depends on traditional medicines to meet their primary healthcare requirements [1] and many of the modern drugs were derived from traditional uses [2, 3]. Factors like accessibility, availability, and cultural acceptability of the healthcare service generally affect the use of traditional medicines [4]. Particularly, in most rural areas of developing countries, medicinal plants serve as major sources of medicines to meet primary healthcare needs [5–7].

Ethiopia is the home for the high diversity of traditional knowledge and practices about the uses of traditional medicine due to the existence of different ethnic groups and complex cultural diversity [8–10]. The practice of traditional medicine in the country is concerned not only with the curing of diseases but also with the protection and promotion of human physical, spiritual, social, mental, and material well-being [11]. The cultivation and use of medicinal plants in Ethiopia have a long history [12] and about 90% of the population use traditional medicine as their first line of healthcare requirements [13]. Furthermore, about 95% of all forms of traditional medicinal preparations are also reported to be of plant origin [14]. The deep-rooted culture of using medicinal plants led the people to be acquainted with the knowledge of medicinal properties of many plants used for treating various human and livestock ailments [15, 16]. However, the rich indigenous knowledge on many of the traditional plant remedies is subjected to loss as it has mainly been passed orally for generations without being properly nor scientifically documented [17].

Despite the significant role played by medicinal plants for treating both human and livestock ailments in Ethiopia, a very limited attempt has been made to explore, document, and promote these widely used medicinal plants in the country. Besides, other factors such as deforestation, overexploitation of natural resources, overgrazing, habitat destruction, and fragmentation, as well as agricultural land expansion, heavily threatened the Ethiopian traditional medicinal plant resource and the associated indigenous knowledge [9]. Thus, it is a timely endeavor to document, promote, and conserve the country's medicinal plant lore. Such documents are important to define and maintain the cultural identity of the people [18] besides providing the opportunity for recognition, promotion, management, and protection of indigenous knowledge of a community on medicinal plants as a vital part of a nation's heritage, establishing people-centered natural resource management system [19] and potentials for scientific discovery of new compounds used in the development of modern drugs [20].

Although Ethiopian medicinal plant inventories including those of [12, 21–28] have attempted to document the importance of traditional medicinal plants in some cultural groups, it is found insignificant when compared to the wide ethnolinguistic communities found in the country, which have remained largely unexplored. Therefore, the present study aims to fill this gap by documenting the wealth of indigenous knowledge on utilization, management, and conservation of medicinal plants used in Kelala District, South Wollo Zone, Ethiopia.

## 2. Materials and Methods

### 2.1. Study Area

**Figure 1:**
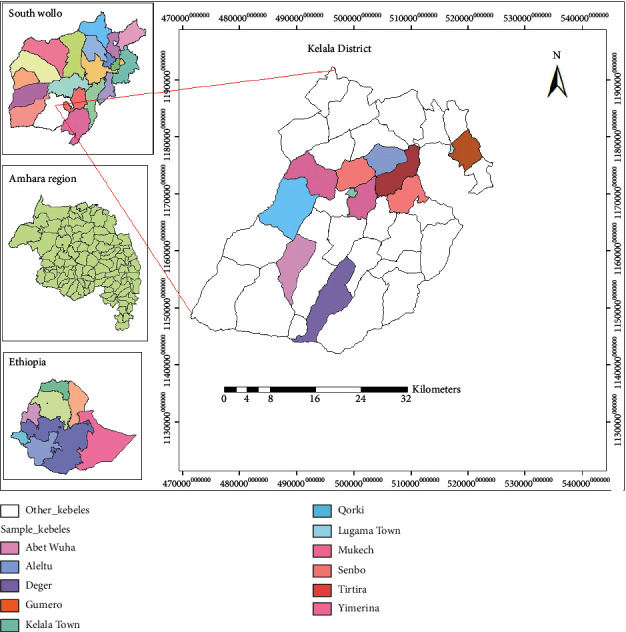
Location map of the study area.

This study aimed to record and document medicinal plants and associated indigenous knowledge of the people in Kelala District, Northeastern Ethiopia. Kelala District is located in the south Wollo Administrative Zone of Amhara Regional State, which is 561 km far away from Addis Ababa. The geographical coordinates of the district lie between 10°28' N and 38°48' E (Figure 1). The district has an altitudinal variation that ranges from 500–2300 m a.s.l. and it covers a total surface area of 143,433 ha [29]. The study area has a unimodal rainfall with a long rainy period from June to October and small rain from March to May [30]. The mean annual rainfall of the study area is 988 mm. The mean annual temperature of the study area is 17°C ranging from the mean annual minimum of 6.2°C to the mean annual maximum of 29.2°C [31].

According to [32], the dominant soil types occurring in the area are clay (17.4%), clay loam (0.6%), and clay to clay-loam (63.5%), loam to clay (0.7%), and silt clay (17.8%). Among these soil types, clay to clay-loam is the most abundant and suitable for cultivation of cereal crops. The vegetation of the study area belongs to dry single-dominant Afromontane forest and this type of forest is known to occur on the plateau of Tigray, Gonder, Wollo, and Harerge regions with an annual rainfall distribution between 500 and 1500 mm. The typical dominant species in the upper storey of these forests is *Juniperus procera* and *Olea europaea* subsp. *cuspidata.* Sometimes the juniper trees can be rather scattered and the forest is characteristic of *Juniperus* woodland with discontinuous evergreen undergrowth [33].

Based on the 2007 national population and housing census, Kelala District had a total population of 136,545 where 67,929 were men and 68,616 women. The majority of the people (94.4%) lives in the rural area by directly obtaining their means of subsistence from agriculture and associated activities while 5.6% of the people are urban inhabitants. More than 95% of the inhabitants are Muslims whereas 4% of the population practice Christianity [34].

In the district, the major ten human diseases are dyspepsia, diarrhea (nonbloody), acute febrile illness, acute upper respiratory infection, infection of the skin and subcutaneous tissue, disease of the musculoskeletal system and connective tissue, pneumonia, urinary tract infection, helminths, and trauma (injury, fracture, etc.). The most important livestock diseases in the district include sheep and goat pox, pasteurellosis (ovine and bovine), mange mites, blackleg, anthrax, African horse sickness, contagious eczema, lice, and fleas' infection, and rabies [35].

### 2.2. Site and Informant Selection

A reconnaissance survey of the study area was conducted from September 20 to October 10, 2017, and resulted in the identification of eleven study sites, namely, Abet wuha, Lugama, Senbo, Kelela, Yimerina rebortu, Deger, Tirtira, Qorki, Aleltu, Gumero, and Mukech. These study sites were selected based on the presence/absence of traditional medicinal practitioners, and the recommendations of knowledgeable elders, kebele administrators, and health workers. Also, other infrastructures such as roads and transportation facilities and agro-climatic zones of the district were considered to select the study kebeles.

A total of 60 (41 males and 19 females) informants were selected from the population following [36]. Out of these, 40 general informants were selected randomly and 20 key informants were selected purposively based on the recommendations of knowledgeable elders, local authorities, and health workers by taking 1-2 individuals from each study kebele. The informants were aged between 20 and 91 years.

### 2.3. Data Collection

Qualitative and quantitative ethnobotanical data were collected from informants using a preprepared semistructured interview method [36, 37]. Focus group discussion, participant observation, and guided field walk were also applied. Ethnobotanical data collection sheet was prepared in English and translated to Amharic language ahead of time to be used during ethnobotanical information retrieval from informants. Information was carefully recorded during an interview with an informant including local names of the medicinal plants, habitat of the plant, disease the plant treats, parts used, methods of remedy of preparation, ingredients added, dosage prescriptions, and routes of administration [36, 37]. Before data collection, written permission was obtained from the culture and tourism office of the district as well as permission from the local administration of each selected kebele. Following this, the purpose of the study was briefly explained to each informant and prior verbal consent was obtained.

#### 2.3.1. Focus Group Discussion and Guided Field Walk

Discussions were conducted to gather further information on medicinal plant knowledge at the community level, ways of transferring their knowledge, major threats on medicinal plants, and indigenous conservation practices. The method of guided field walk with the help of local guides from each study site was applied to make notes on the habit, habitat, morphological features, and association of the medicinal plants with other species. Moreover, it allowed seeing, smelling, and tasting the medicinal plants under investigation to understand the unique features of the species. The local field guides also played a crucial role in identifying the medicinal plants found in the field by proving their vernacular names. Voucher specimen collection was made from the wild and home gardens. Preliminary identification of the specimens was made at the field and the collected specimens were dried, pressed, and taken to the National Herbarium (ETH) in Addis Ababa University. Specimen identification was carried out by using taxonomic keys in the Flora of Ethiopia and Eritrea and comparing with authenticated specimens at ETH.

### 2.4. Data Analysis

Ethnobotanical data were analyzed using basic analytical tools following [36] and descriptive statistical methods such as frequency and percentage. informant consensus factor (ICF) was computed to identify potentially effective medicinal plant species in the respective disease categories [38]. Thus, ICF=(*n*_ur_ − *n*_*t*_)/(*n*_ur_ − 1) , where ICF is informants consensus factor, *n*_ur_ is number of use citations in each category, and *n*_*t*_ is number of times a species used. Preference ranking and direct matrix ranking exercises [36, 39] were computed to recognize use-preference and/or use diversity of medicinal plants by the key informants. Values given by key informants on use-preference and/or use diversity of medicinal plants were added and ranked to get the outputs of the preference ranking and direct matrix ranking. The relative healing potential of each reported medicinal plant used against human diseases was computed as fidelity level (FL) [39]. FL=(*I*_*p* _/*I*_*u*_) × 100, where *I*_*p*_ is the number of informants who independently cited the importance of a species for treating a particular disease and *I*_*u*_ is the total number of informants who reported the medicinal plant for any given disease.

## 3. Results

### 3.1. Diversity of Medicinal Plants

**Figure 2:**
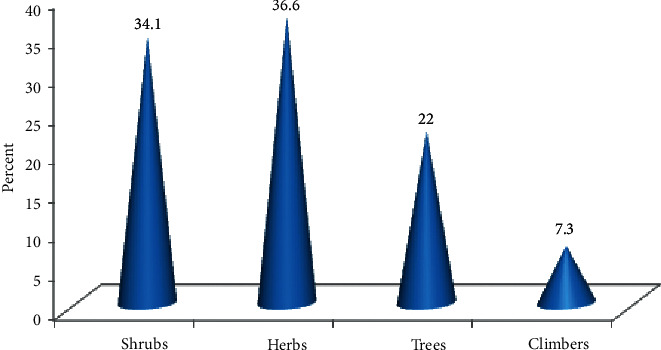
Growth form distribution of medicinal plants.

Eighty-two species of medicinal plants, belonging to 79 genera and 45 families, were used by the local people to treat different human and livestock diseases in Kelala District. Of the total eighty-two medicinal plants, 43 (52.44%) species were used to treat human ailments whereas 33 (40.24%) species were used to treat livestock aliments and 6 (7.32%) species were used to treat both human and livestock ailments. The family Solanaceae had the highest number of medicinal plant representation (seven species, 8.54%) followed by Fabaceae, Asteraceae, Cucurbitaceae, Apiaceae, and Euphorbiaceae (four species each, 4.88%) and Lamiaceae and Poaceae (three species each, 3.67%). Eleven families were represented by two species each (2.44%) whereas the remaining 26 families had single-species representation (1.22%). The growth forms of the medicinal plants indicated that herbs (30 species, 36.6%) had the highest proportion compared to shrubs (28 species, 24.1%), trees (18 species, 22%), and climbers (6 species, 6.5%) (Figure 2). The medicinal plants were harvested from the wild, home gardens, and both the wild and home gardens. Of all the medicinal plants recorded in the study area, large proportions of the medicinal plant species (53, 64.6%) were harvested from the wild followed by home gardens (24, 29.3%), and the remaining species (5, 6.1%) were collected from both the wild and home gardens.

### 3.2. Medicinal Plant Parts Used and Condition of Preparation

**Figure 3:**
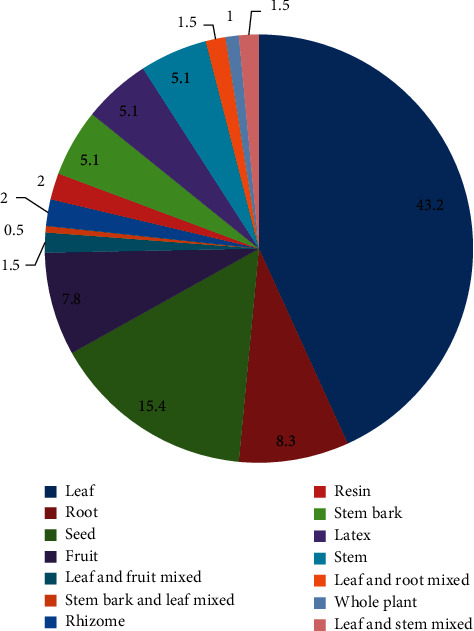
Percentage distribution of medicinal plant parts used in the study area.

**Figure 4:**
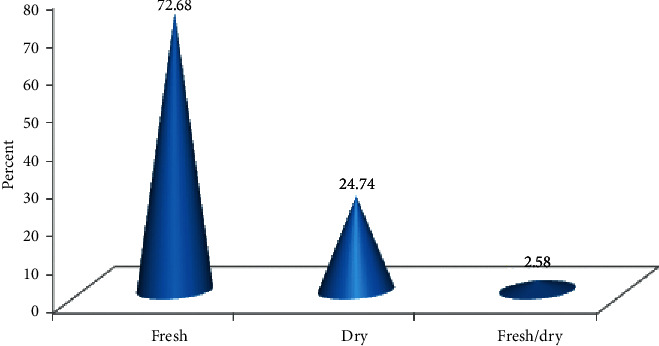
Condition of medicinal plant preparation.

Although the traditional healers mentioned many plant parts to be used for remedy preparation, leaves occupied the highest proportion (42.2%) followed by seeds (15.2%), roots (8.1%), and fruits (7.6%) (Figure 3). Most of the herbal medicine preparation involved the use of a single plant part (95%) whereas the uses of mixing different parts (5%) were not commonly practiced in the study area. The majority of remedies were prepared from freshly harvested plant parts (72.68%) followed by dried form (24.74%) and the remaining (2.58%) were prepared from both fresh and dried parts of medicinal plant species (Figure 4).

### 3.3. Modes of Remedy Preparation, Dosage, and Application

The major modes of remedy preparation from medicinal plant materials were crushing (41.12%) followed by powdering (24.37%) and decoction (13.2%) (Table 1). The traditional healers do not have any standardized doses for herbal remedies prescribed and given to patients. However, approximate dosages were used based on gender, age, pregnancy status, and physical appearance of the patient. Some medicinal plant preparations were measured using small cups (locally called SINI), handful, plastic jug, finger length, and spoon. Different routes of administration of medicinal plant preparations were used to treat various human and livestock ailments in the study area. The most commonly used route of administration was an oral application (52.8%) followed by dermal application (35.5%). Other routes of administration include nasal (3.6%), oral or nasal (3.6%), external (2%), and eye or dermal (1%).

### 3.4. Informant Consensus Factor (ICF)

Results of the analysis of ICF showed that some medicinal plants are effective in treating a certain disease. These medicinal plants that are well known by the informants also have higher ICF values. Accordingly, the category with the highest ICF value was gastrointestinal disorder and parasitic infection (0.77) followed by respiratory diseases (0.70) (Table 2).

### 3.5. The Relative Healing Potential of Medicinal Plants, Fidelity Level

The highest fidelity level (100%) was recorded for *Zehneria scabra* (Linn. f.) Sond. followed by *Ocimum lamiifolium* Hochst. ex Benth. (94.44%), *Impatiens rothii* Hook. f. (92.5%), *Eucalyptus globulus* Labill. (84.38%), *Cordia africana* Lam. (81.25%), and *Otostegia tomentosa* A. Rich. (80%). Medicinal plants with the highest fidelity level scores were found under febrile illness and dermatological disease category (Table 3).

### 3.6. Preference Ranking

Results of analysis of preference ranking on medicinal plants that were reported to be used against stomachache in humans indicated that *Nigella sativa* L. was the most preferred species to treat the disease followed by *Allium sativum* L. and *Zingiber officinale* Roscoe (Table 4).

### 3.7. Direct Matrix Ranking

The results of direct matric ranking exercise on five multipurpose medicinal plants indicated that *C*. *africana* was ranked first followed by *Acacia abyssinica* Hochst ex. Benth. and *Olea europaea* subsp. *cuspidata* (Wall. ex G. Don.) Cif. (Table 5). This finding showed that these species are more exploited for purposes other than their medicinal uses in the study area.

### 3.8. Conservation of Medicinal Plants

The local community in the study area mainly depends on the natural environment for collecting medicinal plants. Despite harvesting the majority of the medicinal plants (53 species, 64.6%) from the wild environment, no remarkable effort was observed to conserve and sustainably utilize the fast-eroding medicinal plant resource of the district. Besides the observed poor experience of cultivating medicinal plants in home gardens, it was reported that most medicinal plants are under threat due to an ever-increasing anthropogenic disturbance on the natural environments of medicinal plants in the area. The major anthropogenic disturbances were agricultural expansion (20.23%), fuel wood collection (19.07%), charcoal production (17.34%), construction (14.45%), and overgrazing (13.87%). Due to the limited accessibility of some medicinal plants, some traditional healers claimed that they have to travel long distances for several hours to collect a particular medicinal plant.

## 4. Discussion

### 4.1. Medicinal Plants of the Study Area

Eighty-two medicinal plants have been documented in this study. The number of reported medicinal plants and plant use knowledge of the local people indicated that Kelala District is rich in medicinal plant diversity and associated indigenous knowledge. In this study, a relatively larger number of medicinal plants were reported compared to some other previous works like 27 species documented in [21], 34 species in [40], 35 species in [41], and 51 species in [42] in different parts of Ethiopia. Out of these reported medicinal plants, 43 (52.44%) species were used to treat human ailments only whereas 33 (40.24%) species were used to treat livestock aliments and 6 (7.32%) species were used to treat both human and livestock ailments. Similar results were reported by [17, 43, 44] in other parts of Ethiopia. The family Solanaceae was represented by seven species followed by Fabaceae and Asteraceae which had four species each. The finding that the family Solanaceae contributed the largest number of medicinal plants in this study agrees well with a similar study conducted elsewhere in Ethiopia [26, 40] whereas various studies in Ethiopia [25, 28, 43] have reported that Fabaceae and Asteraceae are the leading families with the highest number of medicinal plants. This could be attributed to the fact that they are the largest families in the flora area of Ethiopia and Eritrea [45]. This could also be related to its efficient and successful dispersal strategies as well as better adaptation to a wide range of ecological conditions. Some medicinal plants reported in Kelala District were also used as remedies in other parts of Ethiopia. For example, 12 medicinal plants used to treat human diseases and four of the medicinal plants reported to treat both human and livestock diseases in the present study are also reported by [25]. Similarly, 19 medicinal plants reported to treat human ailments in the present study are also reported by [46], suggesting that some of the reported medicinal plants having similar uses in other parts of the country can be taken as an indication of their pharmacological effectiveness [47]. The majority of medicinal plants were collected from the wild environment indicating that the local people have not yet started to cultivate the medicinal plants they are using for medicinal purposes around home gardens and nearby cultivated areas.

### 4.2. Habit of Medicinal Plants and Parts Used

A large number of the medicinal plants 30 (36.6%) collected from Kelala District were herbaceous species. This indicated that most plant remedies were obtained from herbs. This could be attributed to the fact that herbs are more easily available and relatively abundant in the nearby areas than shrubs and trees which are often harvested from forest patches distantly located from their dwelling areas. Besides, herbs occupied the dominant number of species compared to woody plants in the flora area. This finding is in agreement with the general pattern of dominance of herbaceous species observed in many medicinal plant inventories in Ethiopia and elsewhere [21, 25, 43, 48, 49].

This study showed that the most frequently used plant parts are leaves (42.2%) and seeds (15.2%). The usage of leaves for remedy preparation should be promoted as a more sustainable method of accessing plant materials since a certain number of leaves remain on the parent plant to carry on its life functions than the harvesting of the roots which kills the parent plant. It agrees with other ethnomedicinal studies in Ethiopia that reported leaves as the most frequently used plant parts [24, 25, 27, 28, 43]. The traditional healers prepare plant remedies for treating human and livestock ailments from a single plant or plant part. Contrary to the present study, the use of multiple plants or plant parts for a single health problem was reported by previous studies in other parts of Ethiopia [25, 49]. This might be because healers mostly used multiple plants or plant parts to increase the strength and efficacy of the drug.

### 4.3. Mode of Preparation, Dosage, and Route of Application

Crushing was the most widely used method of remedy preparation in the study area. Similar findings were reported by [25, 50, 51] in other parts of Ethiopia. However, the paper [24, 52] reported that powdering and pounding are the dominant method of preparation in Ghimbi and Chelya Districts, respectively. The majority of remedies used for treating various human ailments were prepared from freshly harvested plant parts (72.68%). This could be attributed to the wide-spread traditional belief of attaining high efficacy from fresh remedies due to the higher concentration of active ingredients in the form of secondary metabolites in the fresh plant parts. In addition, traditional healers claim that some medicinal plants lose their healing potential if not used in fresh form. Previous ethnomedicinal works [17, 27] have also indicated the wide use of fresh plant materials for various remedy preparations due to the reportedly better efficacy than when using dried plant materials. The majority of the remedies were administered orally. This finding is in agreement with other studies [27, 28] that reported that oral administration of medicine (70.5% and 52%, respectively) was the leading route of application in other parts of Ethiopia. Even though dosages of remedies for various ailments were reported to be determined based on age, the occurrence of pregnancy, physical fitness/appearance, and gender of the patient, there were no standardized measurements or guidelines set or shared by traditional healers. It was reported that the lack of precise dosage is one drawback of traditional medicinal plants [53,5 4].

### 4.4. Efficacy and the Relative Healing Potential of Medicinal Plants

For this study, the highest ICF values (0.77, 0.70, and 0.67) indicated the best agreement among informants on the use of human medicinal plant species for treating gastrointestinal and parasitic infections, respiratory diseases, and dermatological diseases, respectively. High informant consensus factor values exhibit the presence of strong agreement of informants regarding the therapeutic uses of reported medicinal plant species. Conversely, low informant consensus factor values indicate strong disagreement of informants regarding the therapeutic uses of reported medicinal plant species. Medicinal plants with higher informant consensus values could reflect the important number of use reports for a particular use category. According to [38], high ICF values are important to identify plants of particular interest in the search for bioactive compounds.

Relatively high fidelity level values were reported for *Z*. *scabra* (100%) and *O*. *lamiifolium* (94.44%) against febrile diseases and for *I*. *rothii* (92.5%) against dermatological diseases which could be considered a clue for the high healing potential of these plants against the corresponding diseases. Interestingly, high fidelity level values for the same disease category (febrile illness) have been reported in Ankober District by [23]. Plants with high fidelity level values could also be targeted for further phytochemical investigation to verify the bioactive role inducing high healing results [38, 55]. Furthermore, plants with high FL values could be target species prioritized for conservation, management, and sustainable use after their bioactivities were properly evaluated and confirmed.

### 4.5. Best Ranking Medicinal Plants

Best ranking medicinal plants are priority species for further investigation against stomachache health problems in humans. Ranking of the top five medicinal plants by the seven key informants reveals that *N*. *sativa* stood first followed by *A*. *sativum*, *Z*. *officinale*, *R*. *chalepensis*, and *L*. *sativum*, respectively, as the most-preferred medicinal plants for treating stomachache. This indicates that the aforementioned plant species were found to be culturally acceptable and important in the study area owing to their wide use by a large number of users as well as their curative properties. An ethnobotanical study carried out elsewhere in Ethiopia [46] also reported the use of *L*. *sativum* and *R*. *chalepensis* for treating stomachache. Further investigation of these species for their bioactive ingredients that could be used for treating stomachache may produce promising results.

Direct matrix ranking exercise results showed that a number of multipurpose medicinal plants of the study area including *C*. *africana*, *A*. *abyssinica*, and *O*. *europaea* ranked from first to third, respectively. As indicated in Table 5 of the result section, these plants are being more exploited for their non-medicinal uses than for the reported human medicinal values. Overexploitation of multipurpose medicinal plant species for fuel wood, charcoal production, construction materials, and lumbering purposes were the factors most responsible for accelerating depletion of the species in the study area. Thus, the findings of this study call for urgent conservation measures to save the fast-eroding multipurpose medicinal plant species of the study area. This finding agrees with [56] that reported the same pattern of high exploitation of multipurpose medicinal plants for uses other than their medicinal values in southeastern Ethiopia.

### 4.6. Threats to Medicinal Plants and Conservation Practices

Medicinal plants are highly threatened by the destruction of their habitats and overexploitation of well-known medicinal plants in the study area. Since most medicinal plants were harvested from wild environments, the traditional healers will not have easy access to medicinal plants and they have to travel long distances from their residential area in search of a particular medicinal plant. The effort to conserve medicinal plants by the traditional healers was found to be poor. This could be seen from the low proportion (29%) of medicinal plants harvested from home gardens as compared to those collected from the wild (65%). Traditional beliefs in the area could also have some contributions to the conservation and sustainable utilization of medicinal plants as also reported by other studies [22, 41, 44]. Informants highly agreed that agricultural expansion, firewood collection, and charcoal production are the most threatening factors for medicinal plants in the study area. Similar results were also reported by other studies in Ethiopia [17, 56].

This study provides the first step to record and document medicinal plants' use and the associated indigenous knowledge of local communities in the study area, to relate this knowledge to the conservation status of the species. Promoting in situ and ex situ conservation of medicinal plants in the district as well as assisting their conservation activities with professional guidance helps to abate the fast-eroding of medicinal plants of the study area.

## 5. Conclusion

A considerable number of medicinal plants recorded from the study area imply that Kelala District is a good reservoir of medicinal plant species. Most of these medicinal plants were collected from the wild environment and herbs occupied the largest proportion of medicinal plants to be utilized. The majority of the medicinal plants were harvested for their leaves and the utilization of leaves may not cause serious damage to the plant compared to those plants where their roots are used for medicinal purposes. However, the majority of plant remedies was prepared from fresh plant materials which increases the frequency of using the plant daily. This may cause overharvesting of locally rare medicinal plant species leading to local extinction. Although a large number of medicinal plants have been reported to be used for treating human and livestock ailments in the study area, they are being threatened by different anthropogenic disturbances. Cutting of trees for the expansion of farmlands was the major threat to the vegetation of the area in general and medicinal plants in particular. Combined efforts of both the district agricultural office with other stakeholders is mandatory to stop and/or reduce further loss of medicinal plants in the study area as well as to encourage and support the local communities to grow and conserve medicinal and multipurpose plants species in home gardens and nearby farmland areas.

## Figures and Tables

**Table 1:** Modes of preparation of herbal medicine in the study area.

Modes of preparation	Number of preparations	Percentages
Crushing	81	41.12
Powdering	48	24.37
Decoction	26	13.2
Chewing, taking the sap	11	5.58
Extracting juice, latex	8	4.06
Boiling, inhaling the smoke	7	3.55
Others	16	8.12

**Table 2:** Informant consensus factor values by disease category in the study area.

No	Disease category	No. of species	% of all species	Use citations	% all use citations	ICF
1	Gastrointestinal and parasitic infections	29	35.37	125	25.25	0.77
2	Respiratory diseases	7	8.54	21	4.24	0.70
3	Dermatological	30	36.59	90	18.18	0.67
4	Febrile illness	17	20.73	46	9.29	0.64
5	Livestock parasitic diseases	14	17.07	35	7.07	0.62
6	Livestock diseases	24	29.27	57	11.52	0.59
7	Organ malfunctioning	15	18.29	33	6.67	0.56
8	Internal diseases	15	18.29	31	6.26	0.53
9	Snakebite, scorpion bite	12	14.63	24	4.85	0.52
10	Others	15	18.29	33	6.67	0.56

**Table 3:** Fidelity level values of medicinal plants against a given human disease category.

No	Scientific name	Disease category	*I*_*p*_	*I*_*u*_	FL (%)
1	*Zehneria scabra* (Linn. f.) Sond.	Febrile illness	32	32	100
2	*Ocimum lamiifolium* Hochst. ex Benth.	Febrile illness	34	36	94.44
3	*Impatiens rothii* Hook. f.	Dermatological	37	40	92.5
4	*Eucalyptus globulus* Labill.	Febrile illness	27	32	84.38
5	*Cordia africana* Lam.	Febrile illness	13	16	81.25
6	*Otostegia tomentosa* A. Rich.	Gastrointestinal and parasitic infections	20	25	80.00
7	*Myrtus communis* L.	Dermatological	11	14	78.57
8	*Foeniculum vulgare* Miller.	Internal diseases	7	10	70.00
9	*Artemisia afra* Jacq. ex Willd.	Gastrointestinal and parasitic infections	4	6	66.67
10	*Laggera tomentosa* (Sch. Bip. ex A. Rich.) Oliv. & Hiern.	Gastrointestinal and parasitic infections	7	11	63.64
11	*Nicotiana tabacum* L.	Respiratory diseases	8	13	61.54
12	*Artemisia afra* Jacq. ex Willd.	Respiratory diseases	3	6	50.00

**Table 4:** Preference ranking results of five medicinal plants used for treating stomachache.

Medicinal plants	Respondents (R1–R7)							Total	Rank
	R1	R2	R3	R4	R5	R6	R7		
*Zingiber officinale* Roscoe.	4	3	4	5	4	3	4	27	3rd
*Nigella sativa* L.	4	5	4	5	4	5	4	31	1st
*Allium sativum* L.	4	4	4	4	5	4	4	29	2nd
*Ruta chalepensis* L.	3	3	5	4	4	3	4	26	4th
*Lepidium sativum* L.	4	3	4	4	3	4	3	25	5th

NB: the numbers in the table indicate the ranks given by informants to medicinal plants based on their healing power. The highest score (5) is given for the medicinal plant most effective in treating stomachache and the lowest score (1) is for the least effective plant.

**Table 5:** Average direct matrix ranking score of five key informants for five multipurpose medicinal plants.

Use diversity	*Cordia africana* Lam.	*Olea europaea* subsp*. cuspidata* (Wall. ex G. Don.) Cif.	*Eucalyptus globulus* Labill.	*Acacia abyssinica* Hochst ex. Benth.	*Croton macrostachyus* Del.
Medicine	4	3	2	3	5
Food	1	0	0	1	0
Fuel wood	4	5	5	4	3
Charcoal	2	4	2	5	3
Construction	4	3	5	2	2
Furniture	4	2	3	2	3
Forage	4	3	2	4	2
Total	23	20	19	21	18
Rank	1	3	4	2	5

## Data Availability

The data used to support the findings of this study are included within the article and are also available form the corresponding author on reasonable request.
